# SARS-CoV-2 Orf9b suppresses type I interferon responses by targeting TOM70

**DOI:** 10.1038/s41423-020-0514-8

**Published:** 2020-07-29

**Authors:** He-wei Jiang, Hai-nan Zhang, Qing-feng Meng, Jia Xie, Yang Li, Hong Chen, Yun-xiao Zheng, Xue-ning Wang, Huan Qi, Jing Zhang, Pei-Hui Wang, Ze-Guang Han, Sheng-ce Tao

**Affiliations:** 1 Shanghai Center for Systems Biomedicine, Key Laboratory of Systems Biomedicine (Ministry of Education), Shanghai Jiao Tong University, 200240 Shanghai, China; 2 Advanced Medical Research Institute, Shandong University, Jinan, 250012 Shandong China

**Keywords:** Viral infection, Innate immunity

COVID-19 is caused by SARS-CoV-2.^1^ As of July 16th, 2020, there were 13,579,581 diagnosed cases and 584,782 deaths attributed to COVID-19 reported globally (https://coronavirus.jhu.edu/map.html).^2^ Unfortunately, there is still no effective drug or vaccine for treating this disease. To accelerate drug development, there is an urgent need to identify critical molecular targets and the role they play in infection. Herein, we reported that Orf9b localizes on the membrane of mitochondria and suppresses type I interferon (IFN-I) responses through association with TOM70, and TOM70 overexpression could largely rescue this inhibition. Our results suggest the potential of targeting Orf9b-TOM70 interaction as a novel therapeutic strategy of COVID-19.

Induction of IFN-I is a central event of the immune defense against viral infection.^3^ Upon exposure to RNA viruses, an intracellular antiviral response is initiated by activation of RIG-I like receptors. In particular, when RIG-I/MDA5 detects viral RNA, they trigger a signaling complex on the mitochondrial outer membrane, including the adapter proteins MAVS/TRAF3/TRAF6/TOM70, which ultimately leads to IFN-β production and induction of a host antiviral state.^4,5^ Recent studies have shown that the most prominent feature of SARS-CoV-2, in terms of immune responses as compared to that of other viruses such as influenza A, is that it triggers a very low level of IFN-I.^6,7^ In addition, it has also been found that the chemical, Liquiritin, can inhibit SARS-CoV-2 by mimicking IFN-I.^8^ Thus, understanding how SARS-CoV-2 suppresses IFN-I responses may be a particularly promising approach to devise therapeutic strategies to counteract SARS-CoV-2 infections.

Previous studies have shown that SARS-CoV Orf9b, an alternative open reading frame within the nucleocapsid (N) gene, can significantly inhibit IFN-I production as a result of targeting mitochondria.^9^ In addition, antibodies against Orf9b were present in the sera of convalescent SARS-CoV.^10^ or SARS-CoV-2 patients.^11^ Therefore, we speculate that SARS-CoV-2 Orf9b may play a critical role in coronavirus-host interactions, particularly via an effect on IFN-I production.

To explore the role of Orf9b in host–pathogen interaction, we employed a biotin-streptavidin affinity purification mass spectrometry approach to identify the human proteins that physically interact with Orf9b (Supplementary Fig. 1a). We found that TOM70 scored the highest among all of the identified interactions (Supplementary Table 1). To validate this interaction, we performed co-immunoprecipitation (co-IP) and found that HA-TOM70 co-precipitated with Orf9b (Fig. 1a) and Orf9b could be pulled down with biotinylated TOM70 (Supplementary Fig. 1b). To quantify the binding strength of this interaction, we performed Biolayer Interferometry (BLI) and found that the *K*_d_ is indeed relatively low (44.9 nM) (Fig. 1b).

**Fig. 1:**
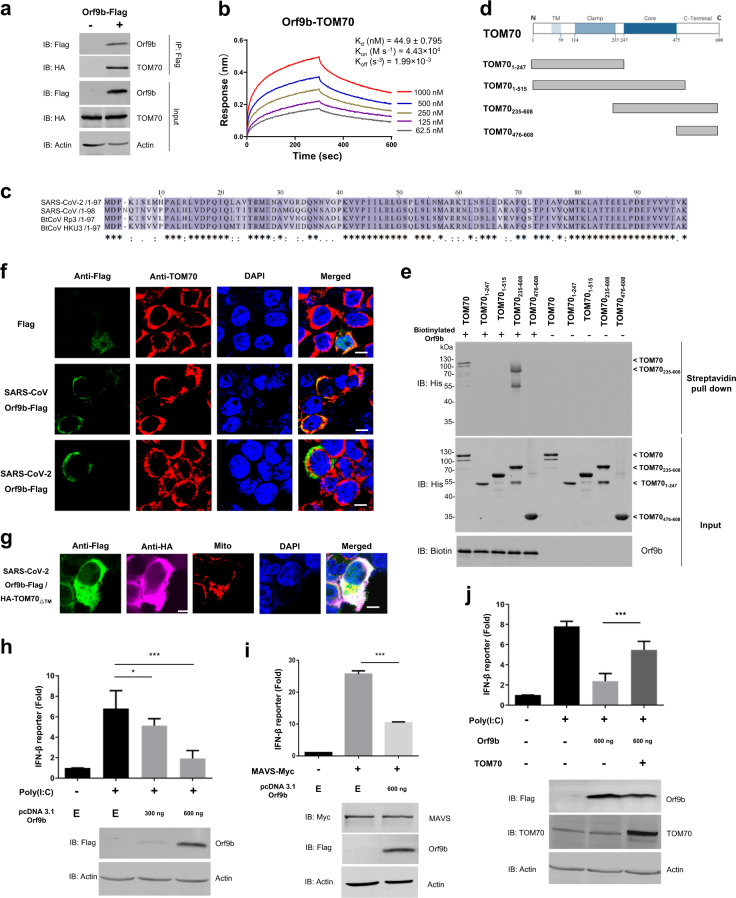
SARS-CoV-2 Orf9b suppresses type I interferon responses by targeting TOM70. **a** Co-immunoprecipitation of Orf9b-Flag with HA-TOM70 from HEK 293T cells. Immunoprecipitation (IP) was performed using anti-Flag magnetic beads. **b** BLI data for the binding of Orf9b to TOM70 and their interaction kinetics. Biotinylated Orf9b was immobilized on streptavidin-coated biosensors and exposed to TOM70 in SD buffer (1× PBS, pH 7.4 with 0.02% Tween-20 and 0.1% BSA). Binding was measured by coincident changes in the interference pattern. **c** Alignment of Orf9b from SARS-like coronaviruses. Sequences were compiled from the National Center for Biotechnology Information server and aligned by means of ClustalW. **d** Schematic drawing of truncated TOM70 used in domain mapping studies. **e** Streptavidin pull down assay was performed by biotinylated Orf9b or BSA incubated with truncated GST-TOM70-His in vitro. **f** Confocal microscopy of HEK 293T cells transfected by SARS-CoV or SARS-CoV-2 Orf9b-Flag, which were stained with an anti-flag antibody (green) and an anti-TOM70 antibody (red). The nuclei were stained using DAPI (blue). Scale bar, 10 µm. **g**. Confocal microscopy of HEK 293T cells transfected by SARS-CoV-2 Orf9b-Flag and HA-TOM70_∆TM_, which were stained with the anti-flag antibody (green) and an anti-HA antibody (magenta). The mitochondria were stained with MitoTracker^®^ Orange CMTMRos (Red) and the nuclei were stained in blue using DAPI. Scale bar, 10 µm. **h**–**j**, IFN-β reporter gene assays using HEK 293T cells expressing Flag or Orf9b-Flag in the presence or absence of HA-TOM70 and induced by transfection of poly(I:C) (**h**, **j**) or MAVS overexpression (**i**). Luciferase activity is shown as fold induction. Data are representative of three replicates (mean and s.e.m. of n = 3 samples), *P < 0.05 and ***P < 0.01 (two-tailed unpaired t-test). **E,** HEK 293T cells expressing Flag only

Considering the high homology of Orf9b in SARS-like coronaviruses (Fig. 1c), we also tested whether SARS-CoV Orf9b interacts with TOM70. Interestingly, we found that SARS-CoV Orf9b exhibits a similar binding strength as SARS-CoV-2 Orf9b, indicating that the interaction may be conserved across the SARS-like coronavirus family (Supplementary Fig. 1c). To further pinpoint the region of TOM70 that is required for the interaction with Orf9b, TOM70 was divided into individual domains according to the known functions of the regions^12^ (Fig. 1d). We found that only the construct consisting of residues 235–608 (TOM70_235-608_) that contained both the core and C-terminal domains precipitated with biotinylated Orf9b, and this interaction was comparable with that of the full-length TOM70 (Fig. 1e, Supplementary Fig. 1d). This suggests that the core and C-terminal domains of TOM70 are essential for this interaction, while the transmembrane and clamp domains are not required.

Since TOM70 is located in the outer membrane of mitochondria, we hypothesized that SARS-CoV-2 Orf9b may also localize to the outer membrane of mitochondria through interaction with TOM70. Indeed, immunostaining of Orf9b-Flag expressing HEK 293T cells revealed that both SARS-CoV and SARS-CoV-2 Orf9b localize to the membrane of mitochondria (Supplementary Fig. 2a) and colocalize with TOM70 (Fig. 1f). Further, we expressed TOM70_∆TM_, a construct without the N-terminal transmembrane domain of TOM70, to investigate whether it would change the mitochondria localization of Orf9b. Despite the presence of endogenous TOM70 in the cells, TOM70_∆TM_ overexpression indeed partially disrupted the association of SARS-CoV or SARS-CoV-2 Orf9b with mitochondria (Fig. 1g, Supplementary Fig. 2b).

Considering the critical role of mitochondria and TOM70 in IFN-I responses,^5^ we next investigated whether Orf9b impacted antiviral IFN-I signaling. We monitored human interferon-β (IFN-β) promoter activity in the presence or absence of SARS-CoV-2 Orf9b using a dual luciferase reporter assay. We observed that Orf9b significantly reduced the activation of IFN-β as compared to that of the vehicle controls. The vehicle controls were prepared by co-transfecting with poly(I:C) (Fig. 1h) or MAVS overexpression (Fig. 1i). Next, we examined whether overexpression of TOM70 can counteract the Orf9b-mediated inhibition of IFN-I responses. We observed that TOM70 overexpression alone could not significantly enhance the expression of IFN-β induced by poly(I:C) (Supplementary Fig. 2c). However, TOM70 overexpression could largely rescue IFN-β expression from Orf9b-mediated inhibition (Fig. 1j). In addition, we also attempted to knockdown TOM70 to further examine the effect of Orf9b on IFN-I through TOM70. However, we did not observe any obvious suppression of IFN-I production upon the addition of TOM70 siRNA (data not shown). We note though an inhibition of IFN-I production by TOM70 siRNA was demonstrated by another study.^5^ While we speculate that these differences may be owing to the differences in the degree of knockdown, further examination is needed to resolve this discrepancy.

Our results thus demonstrate that SARS-CoV-2 Orf9b localizes on mitochondria and suppresses IFN-I responses through association with TOM70. Previous studies have shown that SARS-CoV Orf9b could trigger autophagy in addition to the inhibition of IFN-I responses,^9^ and interestingly, autophagy is also observed upon TOM70 knockdown.^13^ Consistent with our observation, Gordon et. al.^14^ have recently reported that SARS-CoV-2 Orf9b interacts with TOM70, although the functional consequences of this association were not examined. In addition, there is also a preprint article that indicates that SARS-CoV-2 Orf9b, Orf3, Orf6, Orf7a, and Orf7b can suppress IFN-I responses to different extents.^15^

There are two possible explanations how Orf9b inhibits IFN-I responses through interacting with TOM70. First, because HSP90 physically interacts with TOM70 and plays a critical role in the response of TOM70-mediated IFN-I activation,^5^ Orf9b may compete with HSP90 for binding to TOM70. Second, TOM70 may be essential for mitochondrial energy metabolism.^13^ In particular, patients with abnormal TOM70 function suffer from lactic acidosis.^16^ By interacting with TOM70, Orf9b may induce the production of lactic acid, which has been proven to inhibit IFN-I responses.^17^

Considering the critical role of IFN-I in the human antiviral response, restoration of IFN-I production in COVID-19 patients may prove to be a significantly effective therapeutic option. Our results highlight the potential by developing therapeutic agents, which could inhibit the interaction between Orf9b and TOM70 in COVID-19 patients. Further, since SARS-CoV Orf9b is highly homologous to SARS-CoV-2 Orf9b and also binds to TOM70 with high affinity, the same strategy may also be applied to SARS infections.

## Supplementary information

Supplementary Information

## Data Availability

The SARS-CoV-2 Orf9b biotin-streptavidin affinity purification mass spectrometry data are deposited on ProteomeXchange (Project accession: PXD019803). Additional data related to this paper may be requested from the authors.
