# Supplementary material for: SARS-CoV-2 Orf9b suppresses type I interferon responses by targeting TOM70
Source: Cell Mol Immunol. 2020 Jul 29;17(9):998–1000. doi: 10.1038/s41423-020-0514-8 (PMC7387808; doi:10.1038/s41423-020-0514-8)
Supplement: Supplementary file 1 — Supplementary Information [file 41423_2020_514_MOESM1_ESM.pdf]

Supplementary Information

SARS-CoV-2 Orf9b Suppresses Type I Interferon Responses by Targeting TOM70

Jiang *et al.*

Table of contents:

Materials and Methods

Supplementary Figure 1. Orf9b interacts with TOM70.

Supplementary Figure 2. Orf9b localizes on membrane of mitochondria and inhibits type I IFN responses through TOM70.

Supplementary Table 1. The identified interacting protein list of SARS-CoV-2 Orf9b.

Supplementary Table 2. Detailed information of the clones constructed in this study.

Materials and Methods

Cell culture

HEK293T cells were cultured using DMEM supplemented with 10% FBS, and 1% penicillin-streptomycin (Gibco). Transient transfection was performed with lipofectamine 2000 (Invitrogen) following manufacturer's instructions. For SILAC labeling of HEK293T cells, 50 mg of $^{13}C_6$ L-Lys and 50 mg of $^{13}C_6$ $^{15}N_4$ L-Arg were added to 0.5 liter of -Lys/-Arg SILAC DMEM (Thermo) supplemented with 10% FBS and 1% penicillin-streptomycin to generate "Heavy" medium. "Light" medium cells were cultured using DMEM supplemented with 10% FBS, and 1% penicillin-streptomycin.

Construction of expression vectors

The protein sequences were downloaded from GenBank (Accession number: SARS-CoV-2 Orf9b MN908947.3, TOM70 NM_014820.4, SARS-CoV Orf9b NP_828859.1). According to the optimized genetic algorithm[1], the amino acid sequences were converted into *E. coli* codon-optimized gene sequences. Subsequently, the sequences of optimized *E. coli* sequences and original gene sequences were synthesized by Sangon Biotech. (Shanghai, China). The synthesized genes were cloned into pET32a or pGEX-4T-1 and transformed into *E. coli* BL21 strain to construct the transformants. The genes without codon optimization were also synthesized and cloned into pcDNA3.1. All TOM70 variants were constructed by QuikChange® Site-Directed Mutagenesis Kit (Agilent Technologies). For the preparation of the biotinylated protein, the proteins were cloned with a C-terminal Avi-His6 tag into pET32a. Detailed information (the protein tags, the protein expression vectors, optimized gene sequences, and *etc.*) of the clones constructed in this study was given in **Supplementary Table 2**.

Protein preparation

For preparation of the biotinylated proteins, the recombinant C-terminal Avi-His6 proteins and BirA were co-expressed in *E. coli* BL21 by growing cells in 200 mL LB medium to $A_{600} = 0.6$ at 37 °C. Protein expression was induced by the addition of 0.2 mM isopropyl-β-d-thiogalactoside (IPTG)

and 100 μM D-biotin before incubating cells overnight at 16 °C. For the preparation of other proteins, the recombinant proteins were expressed in *E. coli* BL21 by growing cells in 200 mL LB medium to A_{600} = 0.6 at 37 °C. Protein expression was induced by the addition of 0.2 mM isopropyl-β-d-thiogalactoside (IPTG) before incubating cells overnight at 16 °C. For the purification of 6xHis-tagged proteins, cell pellets were re-suspended in lysis buffer containing 50 mM Tris-HCl pH 8.0, 500 mM NaCl, 20 mM imidazole (pH 8.0), then lysed by a high-pressure cell cracker (Union-biotech, Shanghai, CHN). Cell lysates were centrifuged at 12,000 rpm for 20 mins at 4°C. Supernatants were purified with Ni^{2+} Sepharose beads (Smart-lifesciences, China), then washed with lysis buffer (For the purification of biotinylated protein, 1 mM biotin is added to the wash buffer) and eluted with buffer containing 50 mM Tris-HCl pH 8.0, 500 mM NaCl and 300 mM imidazole, pH 8.0. For the purification of GST-tagged proteins, cells were harvested and lysed by a high-pressure cell cracker in lysis buffer containing 50 mM Tris-HCl, pH 8.0, 500 mM NaCl, 1 mM DTT. After centrifugation, the supernatant was incubated with GST-Sepharose beads (Smart-lifesciences, China). The target proteins were washed with lysis buffer twice and eluted with 50 mM Tris-HCl, pH 8.0, 500 mM NaCl, 1 mM DTT, 40 mM glutathione.

Biotin-streptavidin affinity purification mass spectrometry

Cells were harvested and lysed in M-PER® Mammalian Protein Extraction (Thermo) supplemented with 1 mM PMSF. Cells were incubated with Biotinylated Orf9b (Heavy medium) or control (light medium) and streptavidin Dynabeads (Invitrogen) for overnight at 4°C. The beads were then mixed at 1:1 ratio. The mixture was extensively washed with PBS buffer with 0.1% Triton X-100 for six times. Then the mixture was washed with 100 mM ammonium carboxylate. Trypsin (Promega) was used for on-bead trypsin digestion overnight at 37 °C. The digested peptides were collected and cleaned with ZipTips (Millipore) before mass spectrometry analysis. The tryptic peptide digests of the proteins were analyzed on an EASY-nL 1200 system coupled online to a Q Exactive plus mass spectrometer (Thermo Scientific, Bremen, Germany). The peptide sequences were determined by searching MS/MS spectra against the Protein database using the Protein Discoverer (version 2.4, Thermo Scientific) software suite with a precursor ion mass tolerance of 10 ppm and fragment ion mass tolerance of 0.02 Da. Carbamidomethyl (C) was set as

the fixed modification, oxidation (M) and deamidated(NQ) were set as the variable modification.

Co-immunoprecipitation and immunoblot analysis

Cells were harvested at about 48 h after plasmid transfection and then lysed in M-PER® Mammalian Protein Extraction (Thermo) supplemented with 1 mM PMSF. Then cell lysates with/ without Orf9b-Flag were used for immunoprecipitation with Flag Antibody (Millipore) and Protein G beads (Invitrogen). Generally, 1 μg primary antibody was added to 200 μL lysates with 50 μL Protein G Dynabeads (Invitrogen) for 4 h at 4°C. The beads were extensively washed with PBS buffer with 0.1% Triton X-100 and eluted with SDS loading buffer by boiling for 5 min. For immunoblot analysis, the samples were electrophorized using SDS-PAGE and transferred to a nitrocellulose membrane (Millipore). The membrane was probed with indicated antibody and scanned by a LI-COR Odyssey scanner (LI-COR Bioscience).

***In vitro* Streptavidin Pull-down Assays**

Biotinylated bait protein was incubated with candidate binding partners or BSA and streptavidin coated Dynabeads (Invitrogen) in PBS buffer for 4 h at 4°C. The beads were extensively washed with PBST buffer (0.1% Triton X-100) three times and boiled with SDS loading buffer for 5 min.

Luciferase reporter assays

HEK 293T cells were seeded in 24-well plates. On the following day, cells were co-transfected with 200 ng of luciferase reporter plasmid, 20 ng of phRL-TK vector as a luciferase internal control, and the indicated expression vectors with Lipofectamine 2000 (Promega). Empty vector pcDNA3.1 was used to maintain equivalent amounts of DNA in each well. After cultured for 24 hrs, polyinosinic-polycytidylic acid [poly(I:C)] (Sigma) was transfected for 6 h. And cells were harvested to measure the expression of luciferase using a dual-luciferase reporter assay (Promega), using the Synergy™ 2 Muti-Detection Microplate Reader (BioTeks). Each

experiment was replicated three times.

Immunofluorescence staining

HEK293T cells were seeded on gelatin-coated coverslips and transfected with Flag-tagged empty vector, SARS-CoV Orf9b-Flag or SARS-CoV-2 Orf9b-Flag individually. Cells were fixed with 4% paraformaldehyde for 15 min, permeabilized with 0.1% Triton X-100 for 10 min and blocked with 0.5% BSA for 30 min. Cells were immunostained with anti-Flag antibody (Sigma) (1:100) , anti-TOM70 antibody (Proteintech) (1:100) and anti-TOM20 antibody (Abclonal) (1:100) or 500 nM MitoTracker® Orange CMTMRos (Thermo) overnight at 4 °C, followed by incubation with Alexa-488 conjugated anti-mouse antibody (Thermo) (1:1000) and Alexa-647 conjugated anti-rabbit antibody (Thermo) (1:1000) for 1 h. Then 4′,6-diamidino-2-phenylindole (DAPI) was used to localize nucleus. Images were acquired with a confocal microscope (Nikon & A1Si).

Bio-layer Interferometry

For the binding kinetics of SARS-CoV-2 Orf9b with TOM70 or $TOM70_{235\text{-}608}$, biotinylated SARS-CoV-2 Orf9b was loaded at 25 ng/μL in kinetics buffer containing 1×PBS with 0.1% BSA and 0.02% Tween20 onto streptavidin biosensors (ForteBio). Association of TOM70 was tested in kinetics buffer at 62.5, 125, 250, 500, 1000 nM for 5 min, and the association of $TOM70_{235\text{-}608}$ was performed at 275, 550, 1100 nM for 5 min. Dissociation in kinetics buffer was measured for 5 min. For the binding kinetics of SARS-CoV Orf9b with TOM70, biotinylated SARS-CoV Orf9b was loaded at 25 ng/μL in kinetics buffer onto streptavidin biosensors (ForteBio). Association of TOM70 was performed in kinetics buffer at 75, 150, 300, 600, 1200 nM for 5 min. Dissociation in kinetics buffer was monitored for 5 min. BLI assays were carried out in 96-well black plates and analyzed on an OctetRed96 (Fortebio) equipment. Mean K_{on}, K_{off}, K_D values were calculated by a 1:1 global fit model using the Data Analysis software (ForteBio). All the curves were processed using Prism software (GraphPad Prism 6.0).

References:

1. Chutrakul C, Jeennor S, Panchanawaporn S, et al. Metabolic engineering of long chain-polyunsaturated fatty acid biosynthetic pathway in oleaginous fungus for dihomo-gamma linolenic acid production. *J Biotechnol* 2016; **218**: 85-93.

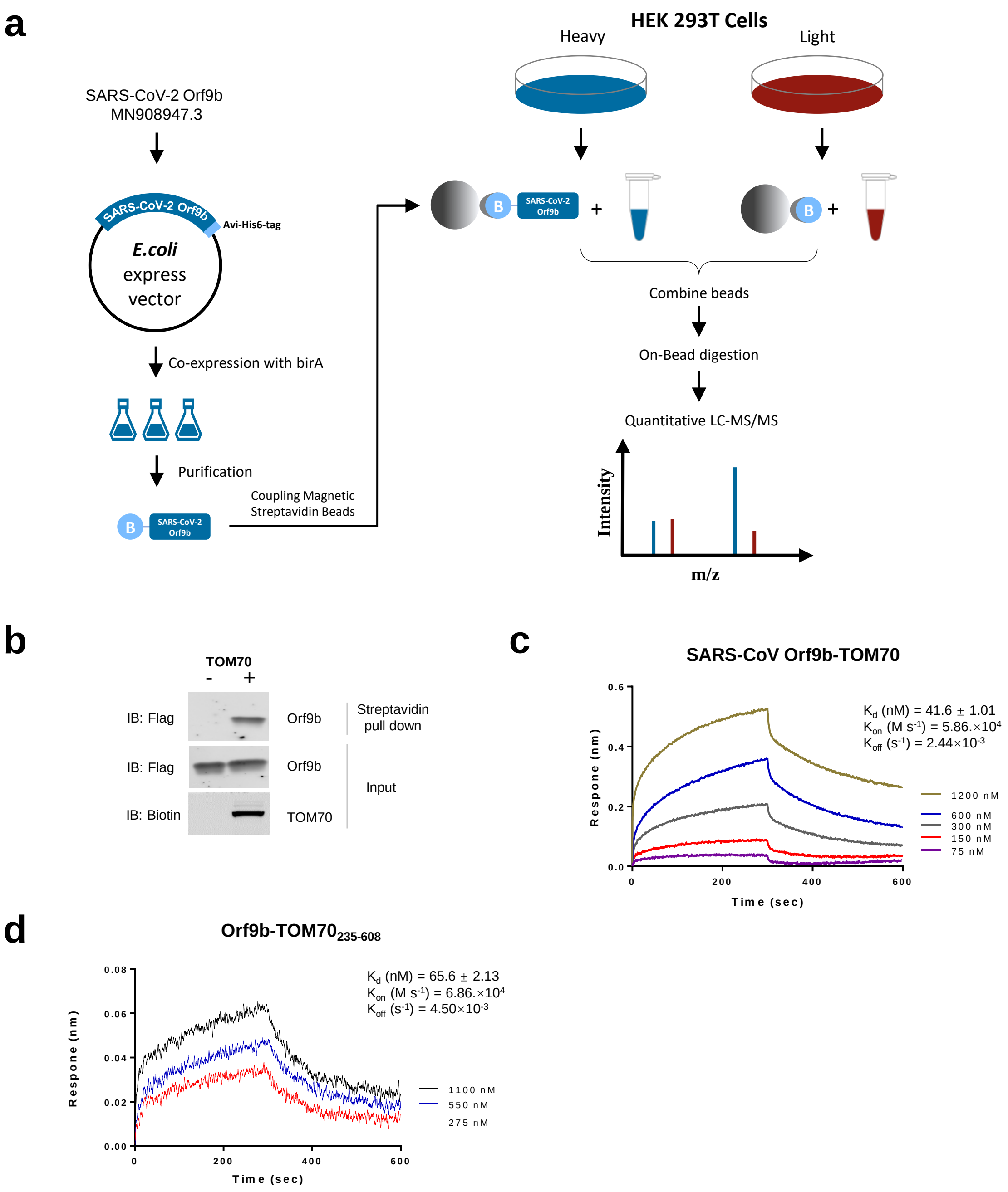

Supplementary Fig. 1 Orf9b interacts with TOM70. a, Workflow of Biotin-streptavidin affinity purification mass spectrometry. **b,** Streptavidin pull down assay was performed by biotinylated TOM70 incubated with BSA or Orf9b-Flag *in vitro*. **c-d,** BLI data for the binding of SARS-CoV Orf9b to TOM70 (**c**) or Orf9b to $TOM70_{235\text{-}608}$ (**d**) and their interaction kinetics. Biotinylated Orf9b was immobilized on streptavidin-coated biosensors and exposed to TOM70 in SD buffer (1 × PBS, pH 7.4 with 0.02% Tween-20 and 0.1% BSA). Binding was measured by coincident changes in the interference pattern.

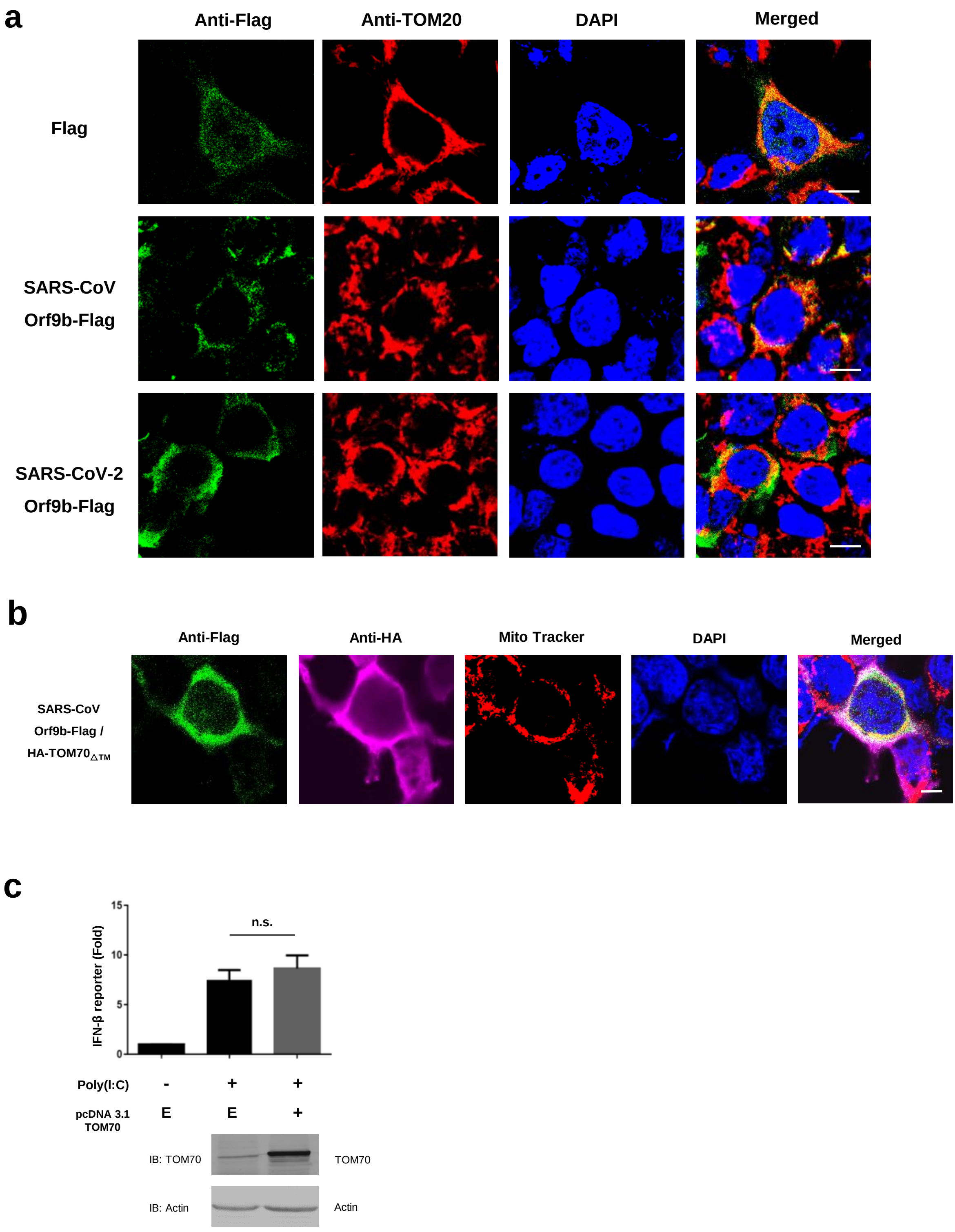

Supplementary Fig. 2. Orf9b localizes on membrane of mitochondria and inhibits type I IFN responses through TOM70. a, Confocal microscopy of HEK 293T cells transfected by SARS-CoV or SARS-CoV-2 Orf9b-Flag, which were stained with anti-flag antibody (green). The mitochondria were stained with TOM20 (red) and the nuclei were stained in blue using DAPI. Scale bar, 10 μm. **b.** Confocal microscopy of HEK 293T cells transfected by SARS-CoV Orf9b-Flag and HA-TOM70$_{\Delta TM}$, which were stained with anti-flag antibody (green) and anti-HA antibody (magenta). The mitochondria were stained with MitoTracker® Orange CMTMRos (Red) and the nuclei were stained in blue using DAPI. Scale bar, 10 μm. **c,** IFN-β reporter gene assays using HEK 293T cells expressing Flag or HA-TOM70 and induced by transfection of poly(I:C). Luciferase activity is shown as fold induction. Data are representative of three replicates (mean and s.e.m. of n=3 samples), n.s., not significant (two-tailed unpaired t-test). E, HEK 293T cells expressing Flag.

Supplementary Table 1 The identified interacting protein list of SARS-CoV-2 Orf9b

Accession	Description	Abundance Ratio: (F1, Heavy Sample) / (F1, Light Control)	Abundances (Grouped): F1, Light Control	Abundances (Grouped): F1, Heavy Sample	PSMs	Unique Peptides	# AAs	MW [kDa]
O94826	Mitochondrial import receptor subunit TOM70	11.594	15.9	184.1	4	3	608	67.4
P29373	Cellular retinoic acid-binding protein 2	7.927	22.4	177.6	3	3	138	15.7
P61224	Ras-related protein Rap-1b	7.248	24.2	175.8	4	3	184	20.8
Q96KR1	Zinc finger RNA-binding protein	7.705	23	177	2	2	1074	116.9
P29966	Myristoylated alanine-rich C-kinase substrate	2.152	63.4	136.6	2	2	332	31.5

Supplementary Table 2. Detailed information of the clones constructed in this study

No.	Protein Name	NCBI GenBank	Vector	Tag	Restriction sites	Expression System
**1**	SARS-CoV Orf9b	Optimized gene 1	pET32a	C-ter-Avi-His6	NdeI/XhoI	*E. coli*
**2**	SARS-CoV-2 Orf9b	Optimized gene 2	pET32a	C-ter-Avi-His6	NdeI/XhoI	*E. coli*
**3**	TOM70	Optimized gene 3	pET32a	C-ter-Avi-His6	NdeI/XhoI	*E. coli*
**4**	SARS-CoV-2 Orf9b	Optimized gene 1	pET32a	C-ter-Flag-His6	NdeI/XhoI	*E. coli*
**5**	TOM70	Optimized gene 3	pGEX-4T-1	N-ter-GST / C-ter-His6	BamHI/XhoI	*E. coli*
**6**	Truncated TOM70	Optimized gene 3	pGEX-4T-1	N-ter-GST / C-ter-His6	BamHI/XhoI	*E. coli*
**7**	SARS-CoV Orf9b	NP_828859.1	pcDNA3.1	C-ter-Flag	HindIII/EcoRI	HEK 293T
**8**	SARS-CoV-2 Orf9b	MN908947.3	pcDNA3.1	C-ter-Flag	HindIII/EcoRI	HEK 293T
**9**	TOM70	NM_014820.4	pcDNA3.1	N-ter-HA	HindIII/EcoRI	HEK 293T
**10**	TOM70$_{\triangle TM}$	NM_014820.4	pcDNA3.1	N-ter-HA	HindIII/EcoRI	HEK 293T

Optimized gene sequences for *E. coli*

Optimized gene 1. SARS-CoV Orf9b

ATGGATCCGAACCAGACCAACGTTGTTCCGCCGGCGCTGCACCTGGTTGATCCGCAGATCCAGCTGACCATCACCCGTATGGAA
GATGCGATGGGCCAGGGCCAGAACAGCGCGGATCCGAAAGTTTACCCGATCATCCTGCGTCTGGGCAGCCAGCTGAGCCTGAG
CATGGCGCGTCGTAACCTGGATAGCCTGGAAGCGCGTGCGTTCCAGAGCACCCCGATCGTTGTTCAGATGACCAAACTGGCGAC
CACCGAAGAACTGCCGGATGAATTCGTTGTTGTTACCGCGAAA

Optimized gene 2. SARS-CoV-2 Orf9b

ATGGACCCGAAAATCAGCGAAATGCACCCGGCGCTGCGTCTGGTTGATCCGCAGATCCAGCTGGCGGTTACCCGTATGGAAAAC
GCGGTTGGCCGTGATCAGAACAACGTTGGCCCGAAAGTTTACCCGATCATACTGCGTCTGGGTAGCCCGCTGAGCCTGAACATG
GCGCGTAAAACCCTGAACAGCCTGGAAGATAAAGCGTTCCAGCTGACTCCGATCGCGGTTCAGATGACCAAACTGGCGACCACC
GAAGAACTGCCGGATGAATTCGTTGTTGTTACCGTTAAA

Optimized gene 3. TOM70

ATGGCCGCCTCTAAACCTGTGGAGGCAGCGGTGGTCGCAGCCGCTGTACCGAGCTCCGGGAGTGGGGTGGGCGGCGGCGGGA
CTGCGGGCCCGGGCACGGGGGGGCTGCCGCGATGGCAGCTGGCTCTGGCGGTCGGGGCACCCCTGCTGCTGGGCGCGGGTG
CCATATACCTGTGGAGTCGTCAGCAGCGTCGTCGTGAAGCGCGTGGTCGTGGTGATGCGTCTGGCCTGAAACGCAACTCTGAAC
GCAAAACCCCGGAAGGCCGTGCGTCCCCGGCACCGGGTAGCGGCCATCCGGAGGGTCCGGGCGCGCACCTGGATATGAACTC
TCTGGACCGTGCACAGGCAGCGAAAAACAAAGGTAACAAATACTTCAAAGCAGGCAAATACGAACAGGCCATCCAGTGCTATACC
GAAGCGATTAGCCTGTGCCCGACCGAGAAAAACGTTGATCTGTCTACCTTCTATCAGAACCGTGCAGCGGCGTTCGAACAGCTG
CAAAAATGGAAAGAAGTGGCGCAGGATTGCACCAAAGCTGTTGAACTGAACCCGAAATACGTTAAAGCGCTGTTCCGTCGTGCTA
AAGCACACGAAAAACTGGATAACAAAAAAGAATGTCTCGAAGATGTTACCGCTGTTTGCATCCTGGAAGGCTTCCAGAACCAGCA
GTCCATGCTGCTGGCAGATAAAGTTCTGAAACTGCTGGGTAAAGAAAAAGCGAAAGAAAAATATAAAAACCGTGAACCACTGATG
CCGTCCCCGCAGTTCATCAAATCTTACTTCTCTTCTTTCACCGATGACATCATCAGCCAGCCGATGCTGAAAGGCGAAAAATCTGA
TGAAGATAAAGATAAAGAAGGTGAAGCGCTGGAAGTGAAAGAAAACTCTGGTTACCTGAAAGCGAAACAGTACATGGAAGAAGAA
AACTACGATAAAATCATCTCTGAATGTAGCAAAGAAATCGACGCGGAAGGCAAATATATGGCTGAAGCGCTGCTGCTGCGTGCTA
CCTTCTACCTGCTGATCGGTAACGCGAACGCGGCTAAACCGGATCTGGATAAAGTTATCAGCCTGAAAGAAGCTAATGTGAAACT
GCGCGCTAACGCGCTGATCAAACGTGGCTCTATGTACATGCAACAGCAGCAGCCGCTGCTGTCTACCCAGGATTTCAACATGGC
GGCTGATATTGATCCGCAGAACGCGGATGTGTACCACCACCGTGGCCAGCTGAAAATTCTGCTGGACCAGGTTGAAGAAGCAGT
TGCGGATTTCGATGAATGCATCCGTCTGCGTCCGGAATCCGCGCTGGCGCAGGCGCAGAAATGCTTTGCGCTGTATCGTCAGGC
TTATACCGGTAACAACTCTTCCCAGATCCAGGCGGCGATGAAAGGTTTCGAAGAAGTTATTAAAAAATTCCCGCGTTGTGCGGAA
GGCTACGCACTGTACGCTCAGGCGCTGACCGATCAGCAGCAGTTCGGCAAAGCGGATGAAATGTACGATAAATGCATCGACCTG
GAACCGGATAACGCAACCACCTACGTTCACAAAGGTCTGCTGCAGCTGCAGTGGAAACAGGATCTGGATCGCGGCCTGGAACTG
ATCTCCAAAGCGATCGAAATCGATAACAAATGCGATTTCGCGTATGAAACCATGGGCACCATCGAAGTTCAGCGTGGCAACATGG
AAAAAGCTATTGACATGTTCAACAAAGCGATCAACCTGGCGAAAAGCGAAATGGAAATGGCGCACCTGTATAGCCTGTGCGACGC
GGCTCACGCGCAGACCGAAGTGGCGAAAAAATACGGCCTGAAACCGCCGACCCTG
